# Human activity monitoring system based on wearable sEMG and accelerometer wireless sensor nodes

**DOI:** 10.1186/s12938-018-0567-4

**Published:** 2018-11-20

**Authors:** Giorgio Biagetti, Paolo Crippa, Laura Falaschetti, Simone Orcioni, Claudio Turchetti

**Affiliations:** DII-Dipartimento di Ingegneria dell’Informazione, Università Politecnica delle Marche, Via Brecce Bianche 12, 60131 Ancona, Italy

**Keywords:** Human activity monitoring, Surface electromyography (sEMG), Accelerometer, Wearable system, Wireless sensor nodes, Healthcare, IEEE 802.15.4, USB

## Abstract

**Background:**

The human activity monitoring technology is one of the most important technologies for ambient assisted living, surveillance-based security, sport and fitness activities, healthcare of elderly people. The activity monitoring is performed in two steps: the acquisition of body signals and the classification of activities being performed. This paper presents a low-cost wearable wireless system specifically designed to acquire surface electromyography (sEMG) and accelerometer signals for monitoring the human activity when performing sport and fitness activities, as well as in healthcare applications.

**Results:**

The proposed system consists of several ultralight wireless sensing nodes that are able to acquire, process and efficiently transmit the motion-related (biological and accelerometer) body signals to one or more base stations through a 2.4 GHz radio link using an ad-hoc communication protocol designed on top of the IEEE 802.15.4 physical layer. A user interface software for viewing, recording, and analysing the data was implemented on a control personal computer that is connected through a USB link to the base stations. To demonstrate the capability of the system of detecting the user’s activity, data recorded from a few subjects were used to train and test an automatic classifier for recognizing the type of exercise being performed. The system was tested on four different exercises performed by three people, the automatic classifier achieved an overall accuracy of 85.7% combining the features extracted from acceleration and sEMG signals.

**Conclusions:**

A low cost wireless system for the acquisition of sEMG and accelerometer signals has been presented for healthcare and fitness applications. The system consists of wearable sensing nodes that wirelessly transmit the biological and accelerometer signals to one or more base stations. The signals so acquired will be combined and processed in order to detect, monitor and recognize human activities.

## Background

Wearable sensors, i.e. sensors that are positioned directly or indirectly on the human body, have become very popular in many application fields such as healthcare, sport, fitness, entertainment, ambient assisted living, surveillance-based security, commerce [1].

They generate signals, e.g. electromyography (EMG), acceleration, electrocardiography (ECG), photoplethysmography (PPG), temperature, that are extremely useful in providing accurate and reliable information on people’s activities and behaviors. With the progress of the signal processing techniques, more and more information has been derived from such biosignals [2–6].

Thus, wearable sensors are revolutionizing our life, social interaction and activities in the same way that PCs have done in the last decades.

In recent years, advances in mobile electronics systems, sensor technologies, signal processing, as well as in communication network protocols have launched a new generation of healthcare systems. Telehealth and fitness monitoring are some examples of an area where integrative research and development in wearable/portable technology are performed. As a result, high-capacity, low-power, low-cost, miniature and lightweight sensors have been embedded into clothes, belts, shoes, sunglasses, smartwatches and smartphones, or positioned directly on the body in order to collect a large amount of data such as body position and movement, heart rate, muscle fatigue, and skin temperature [7, 8].

On the one hand, inertial measurement is the most commonly used method to evaluate the physical activity as it allows to record variations in orientation and easily detect body movements. Thus, many accelerometry-based wearable systems for the pervasive monitoring of activities of daily living (ADL) have been developed. When only one inertial sensor is used to monitor and classify activities, the common location choices are the upper arm, the wrist, the waist, and the ankle [9–12]. Waist-located sensors capture major body motions, but algorithms using waist data can underestimate overall expenditure on activities where the waist movement is uncorrelated to the movement of the limbs, e.g. bicycling or arm ergometry. Similar considerations could be done for recent techniques based on smartwatch and smartphone data [13–16]. However, they are effective in monitoring and classifying activities that involve repetitive body motions, such as running, cycling, lifting weights, walking, climbing stairs [17].

Therefore, in order to better address the problem of detecting human activities, several techniques based on the placement of more accelerometer sensors across the body have been recently proposed [18, 19].

On the other hand, accurate estimation of biometric parameters when the subjects are performing various physical exercises, is often a challenging problem due to the presence of motion artifacts that corrupt the bioelectric signals recorded from subjects’ wrist or arm, such as the surface EMG (sEMG) ones [20]. Referring to this point, data derived from a triaxial accelerometer have been demonstrated to be very useful in reducing motion artifacts [21].

Lightweight wireless sensor devices can be comfortably worn during ADL (including sleep) for monitoring purposes. For elderly people they could be used for the detection of alarm conditions generated by unusual behaviours of the person (not getting up from bed, no activity during a defined time interval) or changes in routinely activities related to psychomotor pathologies. For healthy people they could be used during sports activities, for counting exercise routines and repetitions in order to track a workout routine as well as determine the energy expenditure of individual movements. Indeed, mobile fitness coaching has involved topics ranging from quality of performing such sports actions to detection of the specific sports activity [22].

Recent works have demonstrated how the sEMG signal is very helpful in monitoring person’s body posture, physical performance, and fitness level [8, 22–26]. This is due to the fact that it can be obtained using intrinsically noninvasive measurement devices and is relatively easy to acquire. Indeed, this signal being originated from the electrical potentials generated by contracting muscles [27, 28] can be collected simply by contacting electrodes to the skin surface. It is worth to note that the relatively low amplitude of the sEMG signal requires a carefully designed, high-input-impedance, low-noise amplifier for processing it before its recording [29]. The useful bandwidth for the sEMG signal is typically below 500 Hz, but suffers from possibly severe motion-induced artifacts at frequencies below 5 Hz. These low-frequency artifacts must be rejected by the amplifier, otherwise the gain stages could saturate.

With the above considerations in mind, combining sEMG and accelerometer sensors in a single device allows to obtain all the necessary information for accurately examining muscle activity, force, fatigue, directionality and acceleration that are of essential importance in sports performance evaluation, injury prevention, rehabilitation, and human activity monitoring in general [30–35].

**Fig. 1:**
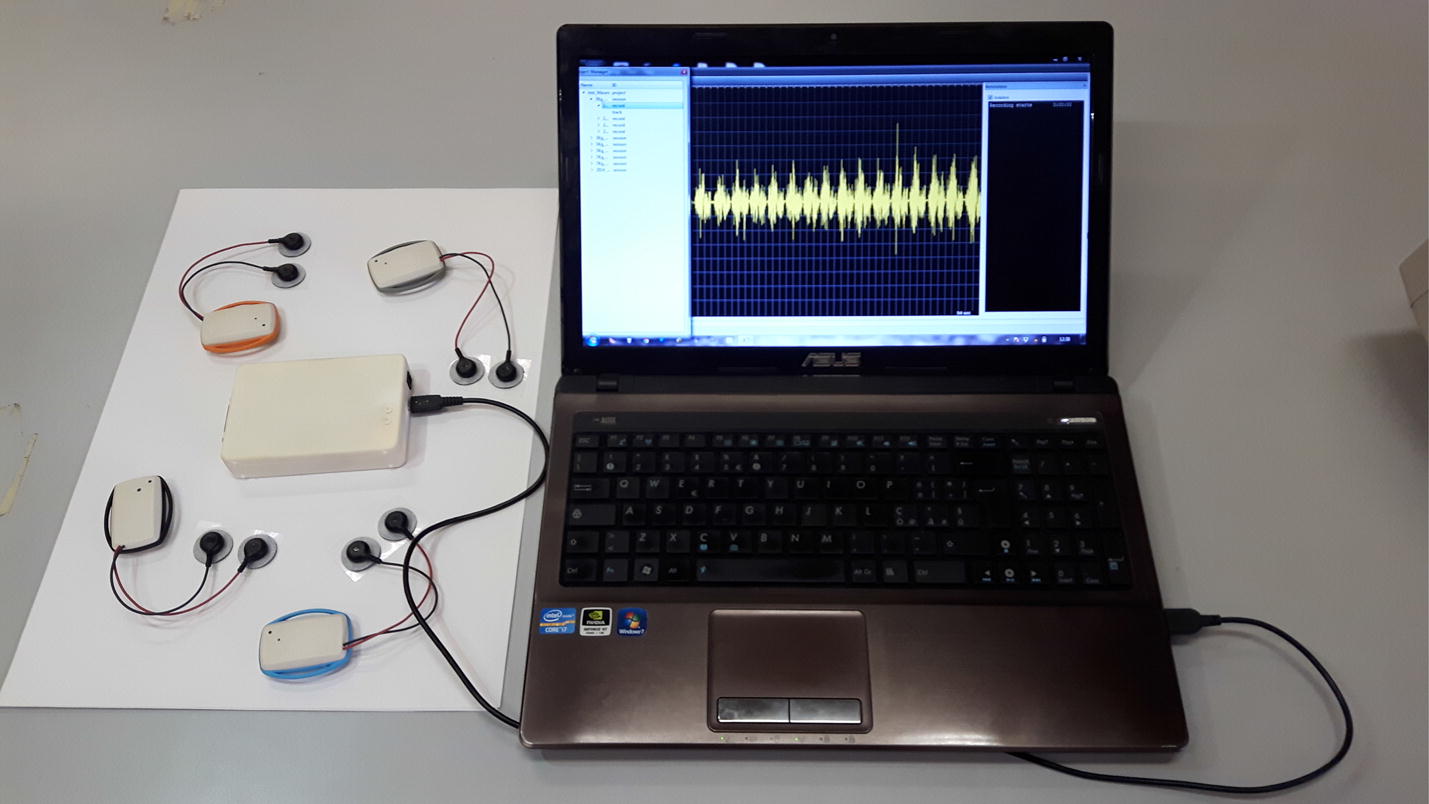
WiSE system. A WiSE system showing one base station connected via USB to the control PC, and four wearable sensor nodes

**Fig. 2:**
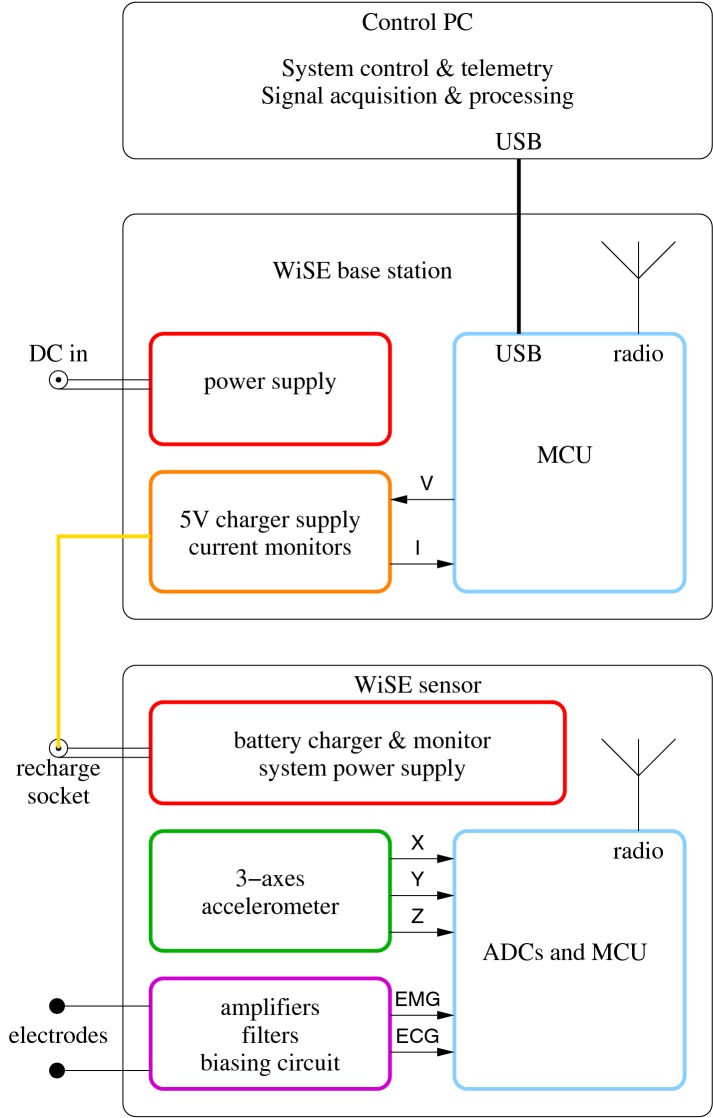
WiSE system block diagram. A view of the three main WiSE system components highlighting the major internal functions

This paper presents a low-cost flexible wireless sEMG system, called WiSE, that is able to acquire both sEMG and motion-related signals using ultralight (23 g) wearable wireless sensor nodes with a software-selectable bandwidth [36]. A photo displaying a set-up of the system, composed by a control PC, a base station and four wearable sensing nodes, is shown in Fig. 1. A more detailed view of the internal blocks is also reported in Fig. 2.

The sEMG/inertial sensing nodes are able to acquire, amplify, digitize, and transmit the signals to one or more base stations through a 2.4 GHz radio link using a custom-made communication protocol designed on top of the IEEE 802.15.4 physical layer, in order to exploit existing low-cost and low-power transceivers but also to enable the possibility of higher throughput and better synchronization than the standard would have allowed. Additionally, the selectable bandwidth allows to easily configure the sensor nodes to capture and process further biological signals such as the ECG signal, as a possibly useful additional feature [37].

The base station can be powered either by an external power supply or by its USB interface, and contains the RF transceiver for the wireless connection to the mobile nodes, a system for simultaneous charging of up to six mobiles nodes, and a 32 bit microcontroller for managing purposes.

A control PC, connected through a USB link to the base station(s), runs a user interface software for viewing, recording, and analysing the data.

Table 1 shows a comparison between the key features of this system and those of several recent research and commercial implementations.

**Table 1:** Key feature comparison of the developed system with similar devices

Device	Signals	Power [mW]	Size [mm^2^]	Weight [g]	# Ch	Sampling Freq. [kHz]
Brunelli et al. [5]	sEMG	96	N.A.	N.A.	1–32	N.A.
Kobayashi [38]	sEMG/ECG	220	N.A.	12	≤ 4	≤ 2
Magno et al. [1]	ECG/sEMG	169.3	N.A.	N.A.	1	0.256
Yousefian et al. [39]	sEMG	16.2	N.A.	N.A.	1–2	2
BITalino [40]	sEMG	13.2	754	2	1	N.A.
BTS FREEEMG [41]	sEMG	N.A.	N.A.	13	20	1
KineMyo [42]	sEMG	N.A.	N.A.	30	≤ 12	1.5625
TRIGNO [43]	sEMG	65	999	14	1	N.A.
This device	sEMG/ECG/ temp./acc.	50	978	23	4	2

In [5] a fully wireless, low cost sEMG acquisition system for prosthetic hand control has been presented. It is capable of acquiring, by using Otto-Bock 13E200 EMG electrodes, up to 32 channels simultaneously, providing adequate bandwidth and signal resolution for complex gesture recognition and prosthetic control.

In [38] a wireless biosignal acquisition system, employing ZigBee wireless technology, has been presented. It consists of two components: an intelligent electrode and a data acquisition host. The active electrode amplifies biosignals such as EMG or ECG and streams the data at up to 2 kSps.

In [1] a wearable wireless low power sensor node which is able to support medical and health care applications is presented. The node is capable of acquiring filtering and amplifying the ECG and the sEMG signals. Then a microcontroller acquires the data via the ADC, runs the respiration and heart rate algorithms and sends them to a host PC through Bluetooth in the EMG and 802.15.4 in the ECG acquisition mode with an overall power consumption of 169.3 mW and 100.7 mW, respectively. The sample rate of the ADC was fixed at 256 Hz, and the host PC used a USB dongle with a coordinator with SimpliciTI protocol for the ECG and a Bluetooth dongle for the EMG transmission.

In [39] the implementations of both one-channel and two-channel sEMG sensors with a low-cost MCU with 2 kHz sampling frequency and 12-bit precision have been proposed.

Finally, in [40–43] four commercial devices capable of acquiring only sEMG signals are also reported.

This paper is organized as follows. After an overall description of the WiSE system, its main features are presented. In order to show how the proposed system could monitor human activity, some results related to the recognition of simple exercises are reported and discussed. Finally, some conclusions end this work.

## System implementation

The WiSE system is composed of the following main components:

- Mobile nodes—these are the signal acquisition units with an embedded wireless transceiver.
- Base station—a USB wireless receiver with integrated charger for the mobile nodes.
- PC software—a user interface software with system diagnostic, signal live-view, recording and analysis capabilities.

The mobile node consists of an active electromyography sensor, a 3-axes linear accelerometer, electrode contact impedance monitoring circuit, and a microcontroller with an integrated wireless transmitter used to digitize the signals and transmit them to the base station connected through a custom protocol. The sEMG sensor, the heart of the system, includes a low-noise and programmable gain stage to adapt the signal acquisition chain to different muscles types. The signal so amplified is then fed into a 10 bit ADC to be digitally transmitted. The accelerometer also has selectable gain, with a full-scale sensitivity of either ± 4 or ± 12 g. Finally, a distributed software-defined phase-locked-loop (PLL) was designed into the protocol to enable the possibility of synchronizing all the nodes to within a microsecond from the base station clock, enabling accurate multi-muscle signal acquisition. More details on the electronical implementation of the system and circuits can be found in [29].

The base station is in charge of coordinating acquisition times and transmission time slots for all the dependent nodes. It is capable of receiving the signals from up to 4 simultaneously active mobile nodes using only a single IEEE 802.15.4 radio channel, thanks to the high bandwidth efficiency of the custom-made radio protocol.

All of the node functionalities, as well as the base station’s, can be remotely monitored and controlled from the same PC by means of a GPL-licensed software that provides full control of the WiSE system. This software provides a user interface that enables real-time acquisition, recording and analysis of sEMG data and system diagnosis. It currently runs under Windows and both Linux x86 and x64 flavors, and provides the following functionalities:

- Support of up to 4 base stations simultaneously connected to the PC and up to 4 active sensors per base station
- Configuration of the mobile nodes for real-time signal acquisition (number of nodes, radio channel, gain)
- Real-time display of acquired data on the PC monitor
- Real-time diagnosis of nodes and base station status
- Data storage in EDF/EDF+ format and plain text files for later processing
- Off-line display of multiple saved tracks and annotations
- Data analysis of stored tracks.

It is structured into three main panels, each one containing its own specific features.

**Fig. 3:**
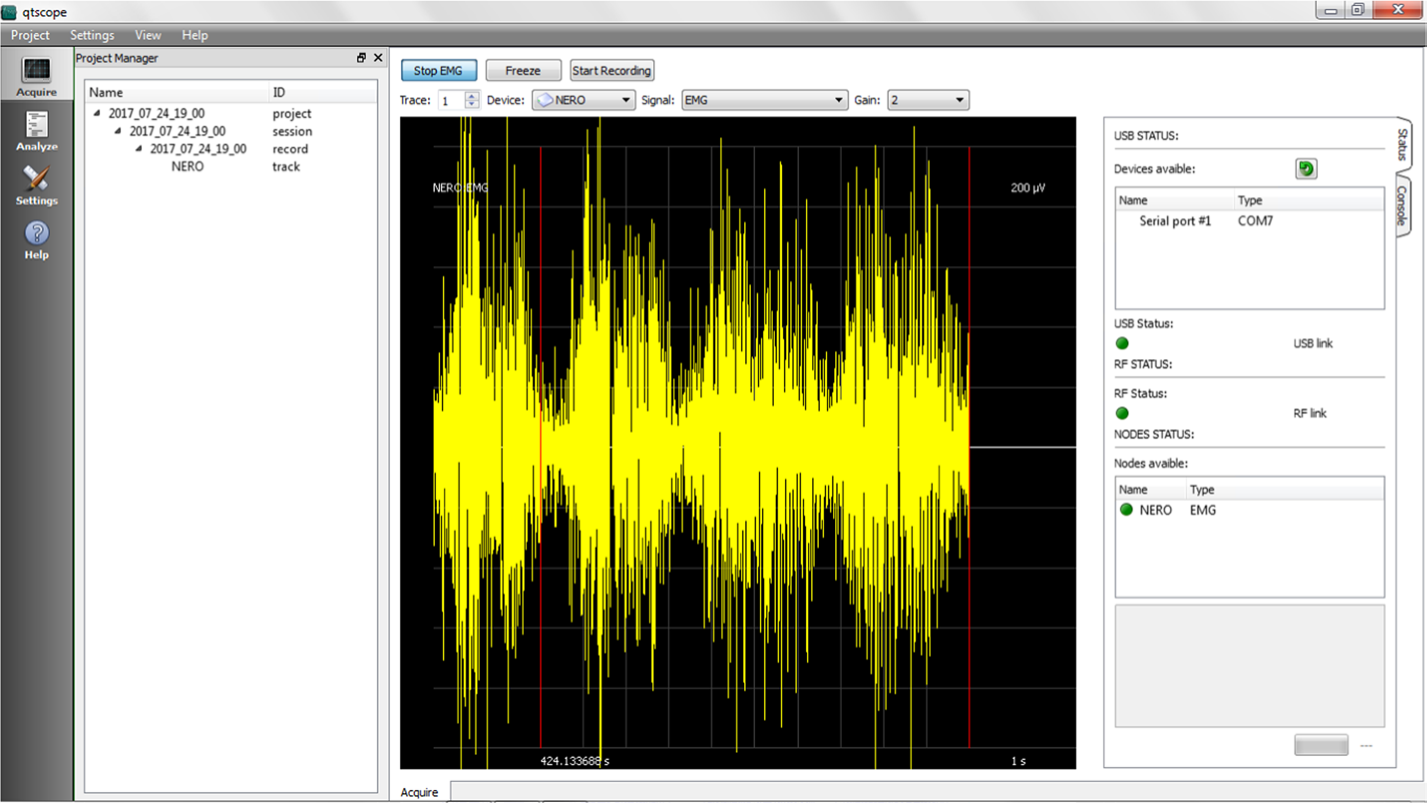
Acquire. Screenshot of acquire function of graphical interface

**Fig. 4:**
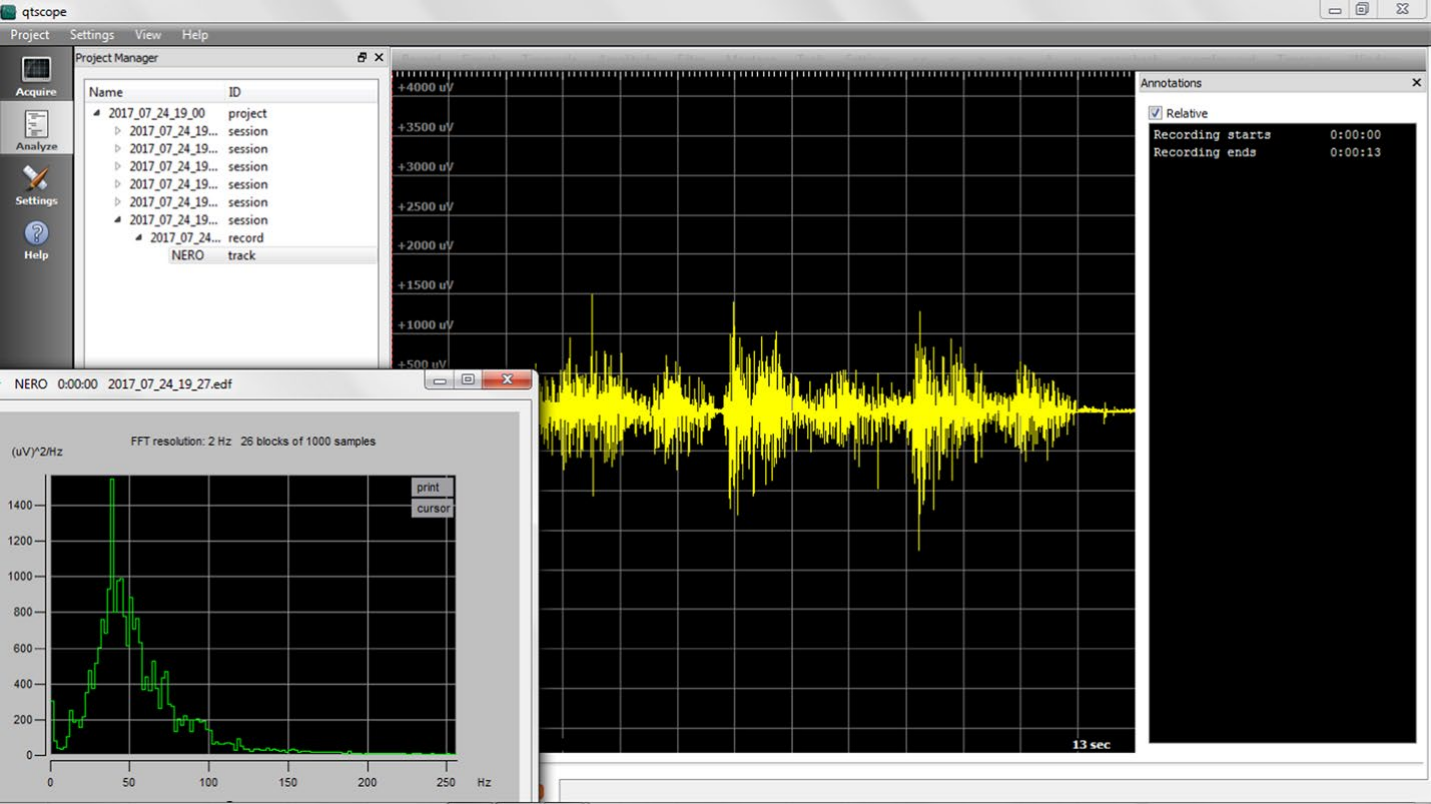
Analyze. Screenshot of analyze function of graphical interface

**Fig. 5:**
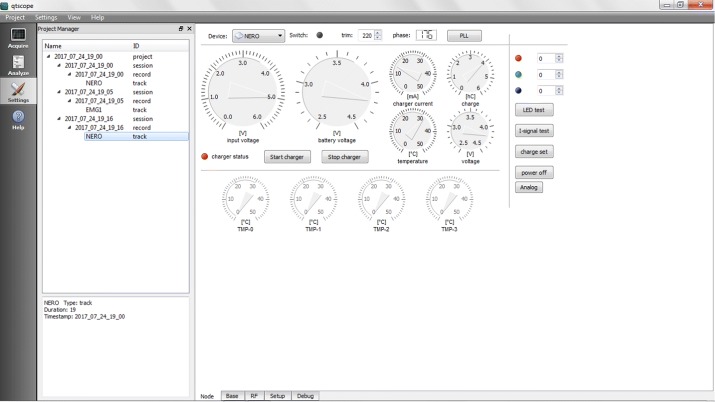
Settings. Screenshot of settings function of graphical interface

1. Acquire: This panel, shown in Fig. 3, allows the automatic start-up of the WiSE system, enabling real-time capture and monitoring of the signal. The PC is responsible for sending, via USB, the commands needed by the base station to wake up the requested nodes, normally in a deep stand-by mode, and to enable them to transmit the acquired data. When the start-up procedure is completed, usually in a few seconds, the measurement starts, and the user can see the real-time traces of the captured signals on the main panel. It is also possible change the gain, number of traces, scale and type of signal to be displayed on-the-fly, and easily manage stored data and metadata. Different scenarios to categorize different measurement setups can also be created.
2. Analyze: In this panel, shown in Fig. 4, the analysis of the signals can be performed. The chosen format for data storage is the European Data Format (EDF), which is a simple and flexible format for the exchange and storage of multichannel biological and physical signals, commonly used also by many commercial devices. This functionality is achieved using *EDFbrowser* and the associated library *EDFlib* [44]; together they provide a C/C++ open source, free and multiplatform framework containing functions for reading/writing and displaying data in EDF format.
  This panel allows the user to open previously saved projects. The signals can then be analyzed with several processing tools (filters, statistical analysis, ...) and the annotations can be edited. For example, in the lower left pane as shown in Fig. 4, the sEMG spectrum can be seen.
3. Settings: This panel, shown in Fig. 5, allows a complete diagnostic of the devices that compose the measurement system to be carried out. The Settings panel contains a “nodes” section and a “base stations” section, so the user can easily select the specific device for which the real-time monitoring of the functional parameters is desired. Specifically, for remote nodes, the software provides several functions such as: charging control, reset, setting MAC addresses and RF channel.

## Methods

**Fig. 6:**
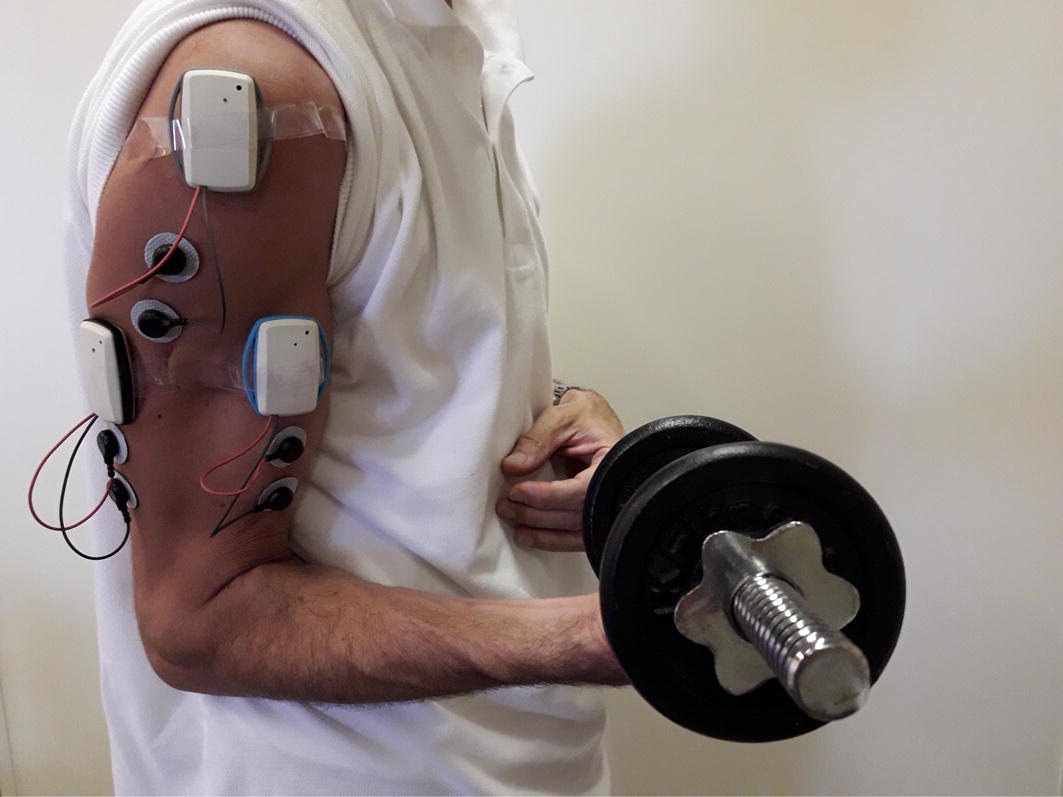
Recording setup. Photograph of the recording setup with the wireless electromyograph sensors worn on the upper right arm with their electrodes placed on the *biceps brachii*, *deltoideus medius*, and *triceps brachii* muscles

To demonstrate the effectiveness of the proposed system in detecting the type of exercise being performed by a subject, a simple experiment was set up. Three sensors were worn by the experimenter on the upper arm, as displayed in Fig. 6. The electrodes were placed on the *biceps brachii*, *deltoideus medius*, and *triceps brachii* muscles by following, for their location and orientation, the SENIAM [45] recommendations. The sensor nodes, which contain the accelerometers, were also aligned along the arm direction, with their “Y” direction parallel to the muscle.

The subjects involved in the experiment where all volunteers who gave their written informed consent in participating in the experiment after having been instructed on the tasks to be performed. In particular, they where asked to perform, according to their fitness conditions, sets of between 10 to 12 repetitions of biceps curls, lateral raises, and vertical raises. In between the latter two, an isometric contraction was to be held for a few seconds. All the exercises were performed with a 3 kg dumbbell.

To better highlight the benefits of the combined approach, an automatic classification experiment was also performed on the data so recorded. The accelerometric signals and the sEMG signal were preprocessed independently to extract relevant features as detailed next, then a KNN classifier was trained using data from two subjects participating in the experiment and tested on the other subjects.

Since the aim is differentiating amongst different motions of the upper arm, the rotation vector was extracted for each node. To this end the accelerometric signals were first low-pass filtered with a cut-off frequency of 0.625 Hz. Let *a*(*t*) be one such filtered signal. From *a*(*t*) we estimate the acceleration due to gravity alone, $\hat{g}$, by averaging it when no movement occurs. Then the signal is sliced into overlapping windows $a_n(t)$ each 8 s long. In each window the extrema of the movement are sought by finding the maxima of the function

1 $$\begin{aligned} d_n(t) = \left\| a_n(t) - \overline{a_n(t)} \right\| \end{aligned}$$

where $\overline{a_n(t)}$ denotes the time average of $a_n(t)$. The maxima of $d_n(t)$ are clustered in two groups corresponding to the endpoints of the movement, and their averages $c^1_n$, $c^2_n$ are computed within these groups. The groups are numbered so that $c^1_n$ is the one corresponding to the rest condition, i.e., the one closest to the estimated $\hat{g}$. The rotation vector $r_n$ is then defined as the normalized cross product between these two averages, multiplied by the angle between them, so that it is expressed in radians:

2 $$\begin{aligned} r_n = \alpha \, \frac{c^1_n \times c^2_n}{\left\| c^1_n \times c^2_n \right\| } , \end{aligned}$$

where $\alpha$ is found by solving the system

3 $$\begin{aligned} \left\{ \begin{array}{l} \sin {\alpha } = k\, \left\| c^1_n \times c^2_n \right\| \\ \cos {\alpha } = k\, ( c^1_n \cdot c^2_n ) \\ \end{array}\right. \end{aligned}$$

It can be noted that the value of *k* is irrelevant in solving the equations, anyway for sake of completeness $1/k=\Vert c^1_n\Vert \,\Vert c^2_n\Vert$.

The processing of the sEMG signal is more straightforward. First, the mean absolute value (MAV) is computed by low-pass filtering the absolute value of the sEMG signal with an identical cut-off frequency of 0.625 Hz. The filtered signal is normalized by dividing it by its average. Let us call *e*(*t*) this signal. As before, *e*(*t*) is sliced in overlapping windows $e_n(t)$ identical to those used for the accelerometric signals. For each window, let $p_n$ be the mean of the peak values of the signal $e_n(t)$. We take as features both the averaged $\overline{e_n(t)}$ and the peak-to-mean ratio $r_n=p_n/\overline{e_n(t)}$.

Finally, these features were tested with an automatic classifier, based on a KNN recognizer.

## Results

**Fig. 7:**
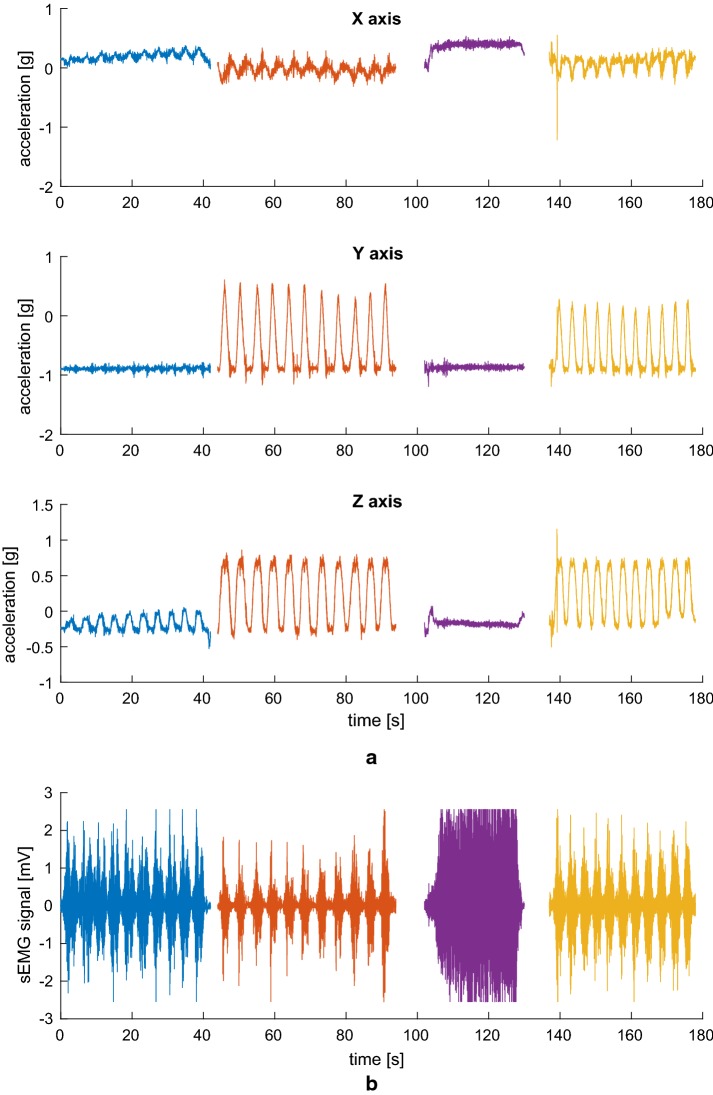
Biceps brachii signals. Three-axis accelerometer signals (**a**) and sEMG signal (**b**) simultaneously acquired from the sensor applied to the *biceps brachii* during the exercise, consisting in 10 biceps curls, 11 lateral raises, an isometric contraction, and 11 vertical raises

**Fig. 8:**
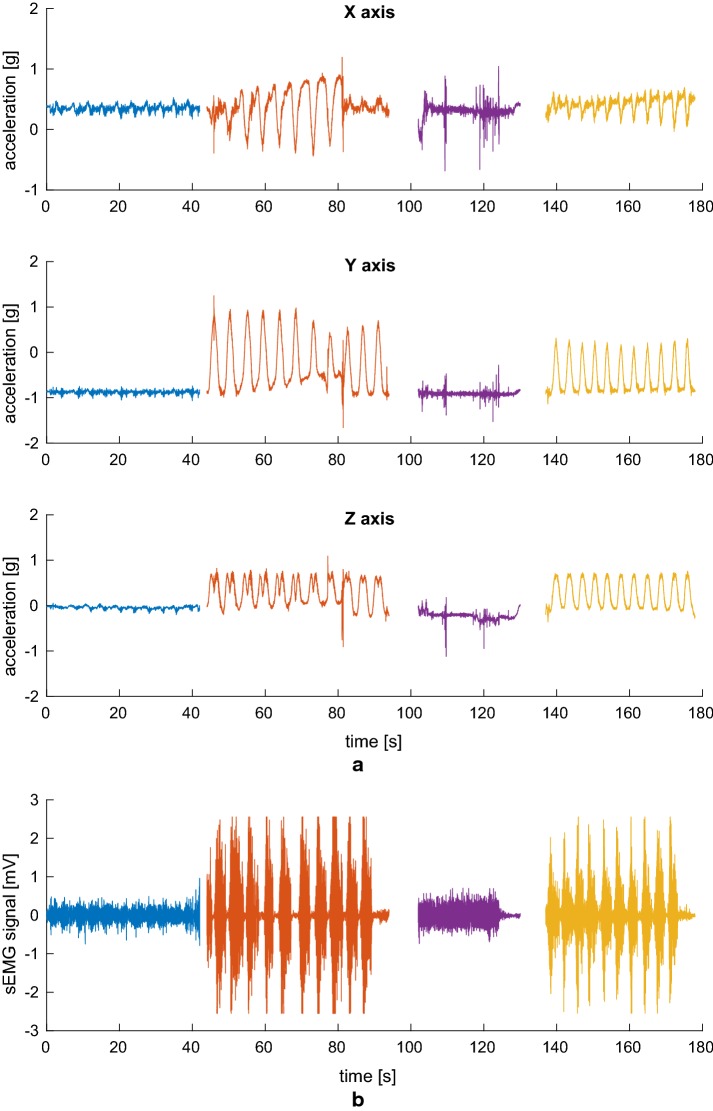
Deltoideus medius signals. Three-axis accelerometer signals (**a**) and sEMG signal (**b**) simultaneously acquired from the sensor applied to the *deltoideus medius* during the exercise, consisting in 10 biceps curls, 11 lateral raises, an isometric contraction, and 11 vertical raises

**Fig. 9:**
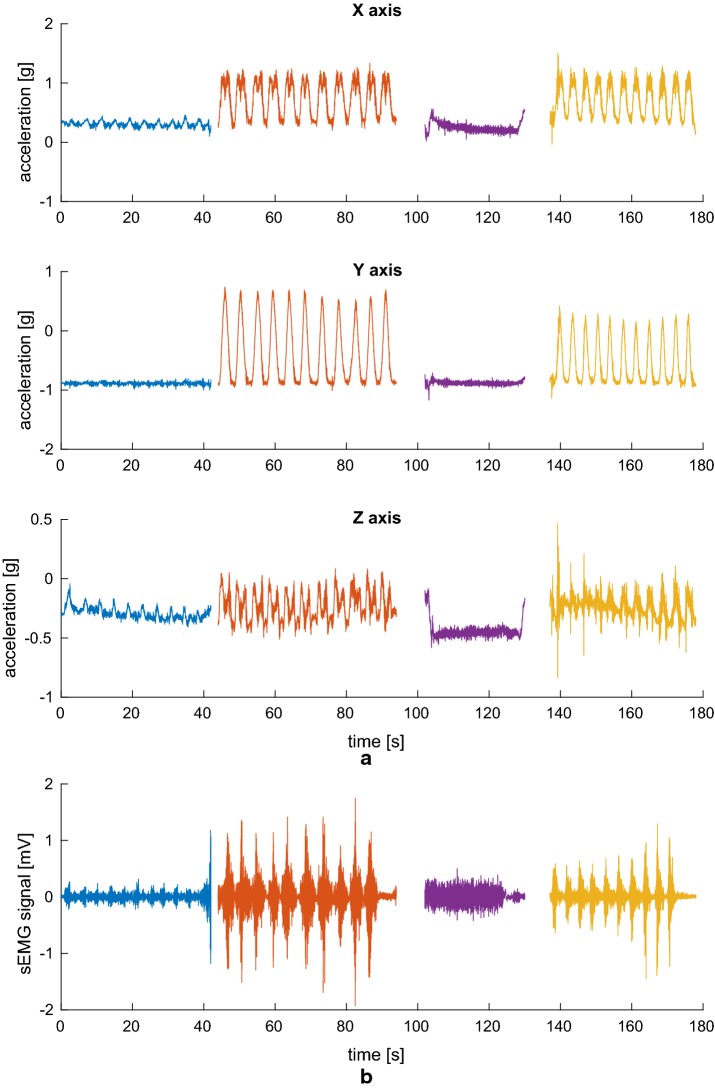
Triceps brachii signals. Three-axis accelerometer signals (**a**) and sEMG signal (**b**) simultaneously acquired from the sensor applied to the *triceps brachii* during the exercise, consisting in 10 biceps curls, 11 lateral raises, an isometric contraction, and 11 vertical raises

For reference, the signals measured on one subject are shown in Figs. 7, 8, and 9.

**Fig. 10:**
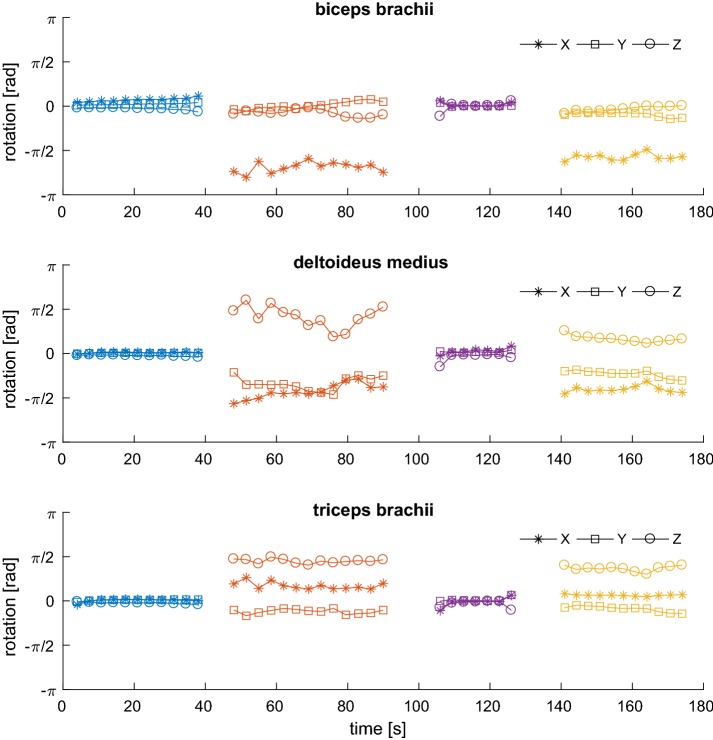
Acceleration-derived features. The components of the rotation vector extracted from accelerometric data from the sensors mounted on the different muscles

Figure 10 shows the rotation vectors extracted from the signals previously reported. It is apparent that the rotation vector is indeed close to zero when the upper arm is still (during biceps curls, first segment, and isometric contractions, third segment), and is close to $\pi /2$ when lateral or vertical raises are performed.

**Fig. 11:**
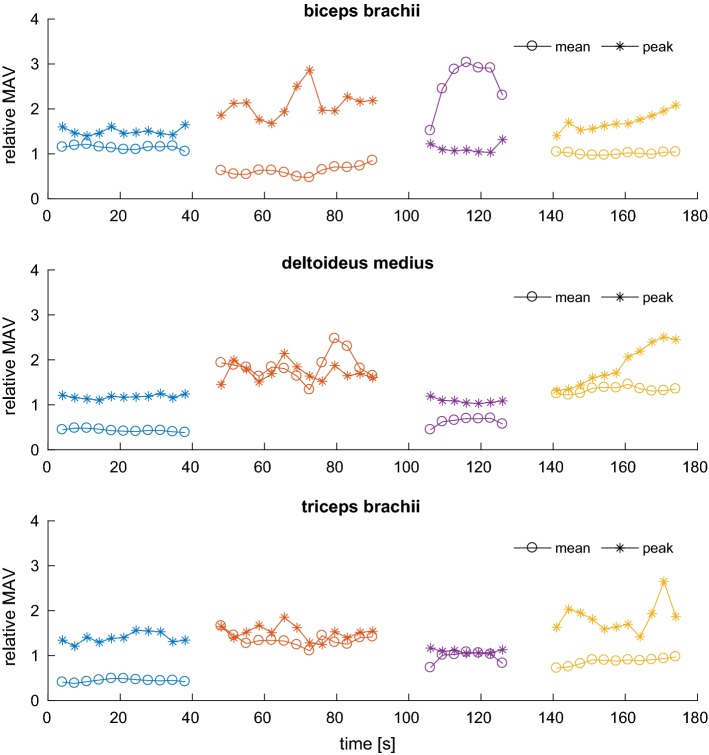
sEMG-derived features. Circles denote the MAV mean within the window, normalized with respect to the whole signal MAV mean, and is thus an index of the average contraction level of the muscle for the specific exercise. Stars denote the ratio between the mean of the peak values within the window and the window mean. It is an index of involvement of the muscle in performing the movement

Figure 11 shows such features for the same signals analyzed before. The most prominent feature is the peak-to-mean ratio $r_n$, denoted with stars. It is apparent that, for the isometric contraction, $r_n$ is very close to unity since the muscle is continuously held flexed, and only somewhat higher for exercises where the muscle never really reaches a rest condition, as in the biceps curls where the *biceps brachii* must always sustain the weight.

Confusion matrices from the experiment are reported in Tables 2, 3, and 4 using accelerometer-derived features alone, sEMG-derived features alone, and all of the features, respectively. Each row of these tables reports the classification results for each kind of exercise, labeled BC for biceps curls, LR for lateral raises, VR for vertical raises, and IM for the isometric contraction. Hence, the column labels represent the estimated exercise types, whilst the row labels are the true ones.

**Table 2:** Recognition with accelerometer data

	BC	LR	VR	IM
BC	0	0	0	23
LR	0	26	0	0
VR	0	10	8	0
IM	2	0	0	15

This table reports the confusion matrix resulting from a classifier trained and tested to only use features extracted from the accelerometers, with an overall accuracy of 58.3%

**Table 3:** Recognition with sEMG data

	BC	LR	VR	IM
BC	23	0	0	0
LR	1	12	13	0
VR	0	9	9	0
IM	3	0	0	14

This table reports the confusion matrix resulting from a classifier trained and tested to only use features extracted from the electromiographic signals, with an overall accuracy of 69.1%

**Table 4:** Recognition with both accelerometer and sEMG data

	BC	LR	VR	IM
BC	23	0	0	0
LR	0	26	0	0
VR	0	10	8	0
IM	2	0	0	15

This table reports the confusion matrix resulting from a classifier trained and tested to use both feature types, with an overall accuracy of 85.7%

## Discussion

As can be seen, availability of the accelerometric signals greatly simplifies the task of detecting the various phases of the exercise, which are highlighted in different colors after having been manually segmented and labeled in Figs. 7, 8, and 9. This would have been much harder to do on the sEMG signal alone, and acceleration information also helps discriminate between the concentric and eccentric phases, an important but complicated task if the sEMG is to be used to evaluate muscle fatigue [27]. Of course, acceleration is not of much use in evaluating the isometric contraction (third segment, in purple), which, on the other hand, clearly stands out in the sEMG track relative to the *biceps brachii* muscle being exerted. It is thus apparent how the combination of the two types of sensors will help in achieving a more complete picture of the activity being performed.

Analyzing the confusion matrix reported in Table 2, it is clear that the accelerometer-derived features alone cannot easily separate BCs from IMs, as in both cases the upper arm is supposed to stay still. This produces a not-very-satisfying recognition overall accuracy of just 58.3%.

These two exercises are instead very well separated using sEMG features, as can be seen in Table 3. Still, the overall accuracy is only 69.1%, because sEMG alone cannot distinguish very well between LRs and VRs.

The situation clearly improves combining the two feature types, as shown in Table 4. Some confusion is still present between lateral raises and vertical raises, as the muscles activation patterns are quite similar and so are the angles the accelerometer can measure, but the combination of the two features is a clear improvement. The system can achieve an overall accuracy of 85.7%, which, considering the somewhat meager training material used, can be considered a good result. We can only suppose that, by increasing the amount of training material, the accuracy will improve.

## Conclusion

In this paper a wireless system for sEMG and accelerometer signal acquisition has been presented for healthcare and fitness applications. The system consists of up to four base stations and several wearable sensing nodes that wirelessly transmit the biological and accelerometer signals to the base stations using a custom protocol based on IEEE 802.15.4 standard. Each base station, that can handle a number of wireless transmitters depending on the type of signal being acquired, is connected via USB to a control PC running a user interface software for data analysis and storage. The custom protocol allowed a high data rate compared to similar devices using the same physical layer and a very precise synchronization, with microsecond resolution, between the different nodes connected to a base station. The signals gathered from the sensor nodes can be combined and processed in order to detect, monitor and recognize the human activity being performed. The experimental results demonstrated that combining the collection of motion and muscle detection data, and integrating their attributes gives a more comprehensive understanding of human activity.
